# Experiences of occupational nurses regarding non-adherent mineworkers with chronic illnesses

**DOI:** 10.4102/hsag.v29i0.2783

**Published:** 2024-09-18

**Authors:** Lorato G. Manyeneng, Mogale L. Pilusa

**Affiliations:** 1 Department of Nursing Science, School of Health Care Sciences, Sefako Makgatho Health Sciences University, Pretoria, South Africa; 2 Adelaide Tambo School of Nursing Science, Faculty of Science, Tshwane University of Technology, Pretoria, South Africa

**Keywords:** medication, non-adherence, chronic illnesses, culture and religious belief, mineworkers

## Abstract

**Background:**

Occupational nurses continue to provide service to mineworkers diagnosed with chronic illnesses, however, non-adherence to medication is prevalent, cause overcrowding, long queues and admission at hospitals as they become sick.

**Aim:**

This study aimed to describe the experience of occupational nurses (ONs) regarding non-adherence to medication among mineworkers diagnosed with chronic illnesses.

**Setting:**

The study was conducted at a selected mine in Gauteng province, South Africa.

**Methods:**

A qualitative, exploratory, descriptive design that is contextual in nature, was used with a phenomenological approach. Thirteen ONs were purposively sampled and individual face-to-face interviews were conducted until data saturation was reached. Thematic analysis using ATLAS.ti 24 software was applied to analyse the data collected. The researcher and the independent coder held a consensus discussion and agreed on the themes and the sub-themes.

**Results:**

Two themes and various sub-themes emerged along with Care-related challenges linked to the mineworkers and challenges related to the provision of service to mineworkers. Data analysed indicated that the ONs had varying but often similar perspectives regarding non-adherence to medication among mineworkers diagnosed with chronic illnesses.

**Conclusion:**

Non-adherence to medication is prevalent among mineworkers diagnosed with chronic illnesses and improving the mineworkers’ outcomes requires addressing the issue of non-adherence to primary medication.

**Contribution:**

This study highlights the importance of adhering to prescribed medication among mineworkers diagnosed with chronic illnesses to ensure quality of life.

## Introduction

Adherence to prescribed medication among mineworkers diagnosed with chronic illnesses is crucial for achieving appropriate and satisfactory clinical results for their conditions. Despite the availability of inexpensive, free and effective treatments in public clinics and hospitals, non-adherence is globally still a common problem. Cheen et al. (2019:2) define the non-adherence to medication as the inability to collect new prescribed treatment that leads to unfavourable clinical conditions and economic effects. Stewart, Moon and Horne (2023:727) indicated that non-adherence is a substantial impediment to achieving optimal results from prescription medications, particularly among individuals diagnosed with chronic conditions. According to Kagee (2004:26) and Sustersic et al. (2019:6) non-adherence of medication is more common in individuals diagnosed with chronic diseases compared with those with acute conditions.

Previous studies have indicated that South African mineworkers who did not adhere to medication reported poorer levels of adherence motivation and self-efficacy, difficulty sustaining a healthy lifestyle, and major issues in keeping control over their lives (Bhagwanjee et al. 2011:357). Muzigaba (2017:1) alluded that non-adherence is prevalent among South African mineworkers. It is evident that non-adherence of medication is common among mineworkers. In Hong Kong, employees in the mining sector choose not to adhere to taking their medications as prescribed, which results in their conditions deteriorating and having secondary complications (Yu et al. 2023:5). Individuals who are diagnosed with chronic illness and choose to discontinue or not adhere to their prescriptions cause overcrowding and long queues at health facilities as they become sick and at times requires admission at hospitals.

Chronic illnesses and diseases are defined differently by various researchers; however, the meaning is the same. Chronic illnesses tend to be of long duration and are mostly as a result of a combination of genetic, physiological, environmental and behavioural factors (Bernell & Howard 2016:2). While The United States National Centre for Health Statistics (USNCHS n.d.) stated that chronic diseases persist for a period longer than 3 months and more, the World Health Organization (WHO 2020) alluded that chronic diseases cannot be transmitted from person to person, they have long duration and generally have slow progressions. The common chronic illnesses that most employees globally find themselves diagnosed with are diabetes mellitus type 1 and 2 [T1D & T2D], hypertension, epilepsy and asthma. Sánchez-Jiménez et al. (2018:1) suggested that most employees are at an increased risk of chronic illness such as diabetes, hypertension and coronary heart diseases. Globally, chronic illnesses trigger non-productivity and high incapacity leave rates at workplaces (Naidoo, Naidoo & Hariparsad 2016:61). In addition, as the population ages, the prevalence of chronic diseases continues to increase (Stewart et al. 2023:726) resulting in some individuals having to take medication.

### Problem statement

Mineworkers are exposed to dangerous working environment, and their conditions need to be monitored and managed to ensure they are healthy and fit to work productively. If mineworkers do not adhere to medication, they increase the chance of treatment failure. Moreover, individuals who do not adhere to medication and manage their health issues jeopardise their finances and quality of life. When chronic conditions are not managed well and controlled, Aaviksoo, Baburin and Kiivet (2013:156) indicated that employees tend to be sick and overuse the sick leave especially in countries with more sick leave benefits. Furthermore, the health system become strained and overburdened by sick individuals who require hospital admission when they do not adhere to treatment (Mekolle 2022:7).

### Research purpose

The purpose of this qualitative synthesis was to describe experiences of occupational nurses (ONs) regarding non-adherence to medication among mineworkers diagnosed with chronic illnesses, at a selected mine clinic in Gauteng province, South Africa.

## Research methods and design

### Research design

A qualitative descriptive phenomenological design was used to describe and make sense of the experiences of ONs regarding non-adherence to medication among mineworkers diagnosed with chronic illnesses. This phenomenological inquiry originates from the psychology and philosophy whereby the researcher describes the lived experiences of individuals about a phenomenon as described by the participants (Creswell & Creswell 2022:14).

### Setting

The study was conducted at a selected mine in Gauteng province, South Africa. The mine has four clinics that provide primary healthcare and occupational health services to mineworkers. The mine was established in 1950 and has approximately 45 000 employees. The mine recruits employees nationally and internationally from countries such as Botswana, Zimbabwe, Lesotho, Mozambique, Malawi, Namibia and Swaziland. There were about 14 000 mineworkers who were diagnosed with chronic illnesses and some of them had their chronic illnesses uncontrolled.

### Population

The total ONs for the clinic were estimated to be 21. The working experiences of ONs at the mine clinic varied from approximately 2 years to 19 years, and most of them had vast experience in managing chronic illnesses among mineworkers.

### Sample and sampling methods

Occupational health practitioners were purposively sampled at the clinic from the target population after they were asked some questions to ensure they fitted the inclusion criteria (LoBiondo-Wood & Haber 2018:106). Before the interviews commenced, they agreed to participate in the study and a consent form was signed. The sample size was 13 which was justified by Creswell and Creswell (2022:268), who stated that saturation occurs when a qualitative researcher stops collecting data because no new data generates new insights or discloses new features.

### Inclusion criteria

The inclusion criteria for the study were that the participants must either be an enrolled or registered nurse, health professional or a medical practitioner registered with the South African Nursing Council (SANC) or the Health Professions Council of South Africa (HPCSA) and had to be involved in treating mineworkers diagnosed with chronic illnesses and they had to have worked at the mine clinic for at least a year or more.

### Exclusion criteria

The exclusion criteria for the study were an enrolled or registered nurse, health professional or a medical practitioner registered with the SANC or the HPCSA those with less than 1 year experience and practitioners who were not involved in management of mineworkers diagnosed with chronic illnesses.

### Pretesting of the data gathering tool

An English interview guide was self-developed by the researcher as a guide to interviews in this study. Pretesting of the tool was conducted on two ONs before the commencement of actual data gathering. Both interviews were audio recorded using a tape recorder and were listened to by both researchers. No flaws were discovered during the interviews and the participants managed to answer all questions satisfactorily. The researchers agreed to approve the interview guide and it was finalised and was applied in the main study.

### Data gathering procedures and tool

Prior to data gathering, the study was approved by the Tshwane University of Technology Ethics Committee and written permission was also acquired from the mine where the study’s participants were employed. The collection of data was arranged with the occupational medical practitioner (OMP) who is the manager at the clinic and who provided the boardroom of the clinic for all interviews conducted. No names of participants were published in any document to ensure and maintain confidentiality and privacy. The consent form was given to each participant prior to the interview to sign after they verbally agreed to participate in the study. The researcher did not know and had never met the participants before the day of data collection. An English interview guide was self-developed after reviewing current literature and theoretical frameworks related to the research subject aids in finding significant themes and topics to be investigated. It had one main trigger question requesting participants ‘what are your experiences as occupational health practitioner regarding medication non-adherence among mineworkers diagnosed with chronic illness’. Face-to-face in-depth individual interviews were conducted, at a place and time agreed by the participants, field notes were taken, and audio tape was used for recording. Open-ended questions were used to direct the interview as well as obtaining lived experiences described by the participants (Fain 2017:205). The interviews were conducted from November 2023 to January 2024, and each interview lasted between 45 and 60 min and were transcribed verbatim by the first author. During the interview, probing was used for more clarification. Information redundancy was reached on the ninth participant (Polit & Beck 2019:62); however, other participants were interviewed as they had agreed to participate in the study.

### Data analysis

Each interview was audio recorded and transcribed verbatim by the researcher. Repetitive listening of audio-recordings and re-reading of transcripts, which were typed on word by the researcher, was performed to develop high quality inductive coding of the participants. The author independently read the interview transcripts to generate ideas for themes and sub-themes. The analysis process began with open coding. This type of coding involves grouping events, objects and actions into categories. Thematic analysis using ATLAS.ti 24 software was applied to analyse the collected data, where two overarching themes emerged from the narrations of participants. Data were analysed using a phenomenological theory approach where smaller portions of data were manually arranged and then imported to ATLAS.ti 24 qualitative data analysis software. Both supervisor and co-supervisor agreed on the coding process.

### Measures to ensure trustworthiness

To ensure the trustworthiness of the study, credibility, confirmability, dependability and transferability were adhered to. Credibility was ensured as the researcher had prolonged presence during interviews with participants for 45 min to 60 min. In addition, the researcher requested the participants to give examples to the answers they gave. Moreover, adequate time was spent at the field to gather or know the social location, culture, and phenomenon of relevance. Furthermore, tests for disinformation and trust building were performed to get to know the data and gain rich information. Prolonged engagements by interviewing and probing participants were done. Research steps were described as well as keeping an audit trail to maintain confirmability and dependability. Transferability was ensured as data gathering was performed until saturation was reached with 13 participants.

### Ethical considerations

The Tshwane University of Technology Ethics Committee approved the study and ethical clearance (FCRE 2022/11/006 [SCI] [FCPS 02]) was obtained. Permission to conduct the research was also acquired from the mine where the study’s participants were employed. The lead researcher established rapport with participants by introducing themselves, explaining procedures, and answering questions prior to data collection. Informed consent was obtained before each interview. Each participant provided permission to audio-record the interviews, and the rationale for taking handwritten notes was explained. To maintain privacy and secrecy, participants’ identities were not published in the research reports, and interviews were conducted in a private room. The study’s inclusion and exclusion criteria were clearly explained, allowing for effective auditing of the acquired data. Each participant was informed that their participation in the study was entirely voluntary and that they might leave at any moment without penalty or consequence.

## Results

The research results presented include the experiences of ONs regarding non-adherence to taking medication among the mineworkers diagnosed with chronic illnesses at a selected mine clinic.

Two overarching themes and various sub-themes emerged from the data analysis. Theme 1 was care-related challenges linked to the mineworkers and theme 2 was challenges related to the provision of service to mineworkers.

### Biographical information

Table 1 outlines the biographical information of participants, which includes their years of work experience at the mine clinic, registration with regulatory professional bodies and their highest academic qualifications. The participants who took part in the study were 13 whereby 9 were females and 4 were males. Furthermore, among the 13 respondents, 8 participants had speciality in their qualification, while 5 had basic qualifications. The years of experience of participants as ON’s at the mining sector is as follows: 9 participants had less than 5 years of experience, 1 participant had more than 5 years of experience, while 3 participants had more than 10 years of experience in the mining sector.

**TABLE 1:** Biographical information of participants.

Job or role in the clinic	Highest qualification	Gender	Regulatory body	Years at the mine clinic
Registered Nurse	Bachelor of Occupational Health Nursing	Female	SANC	19
Registered Nurse	Advanced Diploma in Clinical Nursing Science	Male	SANC	4
Registered Nurse	B Tech Primary Health Care	Female	SANC	6
Registered Nurse	B Tech Primary Health Care	Female	SANC	16
Registered Nurse	B Tech Primary Health Care	Female	SANC	3
Enrolled Nurse	Enrolled Nursing Certificate	Female	SANC	4
Registered Nurse	Diploma in General Nursing	Female	SANC	3
Registered Nurse	B Tech Primary Health Care	Female	SANC	4
Social Worker	Bachelor of Social Work	Male	HPCSA	3
Enrolled Nurse	Enrolled Nursing Certificate	Female	SANC	3
Registered Nurse	Bachelor of Occupational Health Nursing	Female	SANC	15
Registered Nurse	Diploma in General Nursing	Male	SANC	2
Enrolled Nurses	Enrolled Nursing Certificate	Male	SANC	2

HPCSA, Health Professions Council of South Africa; SANC, South African Nursing Council.

### Themes

Two overarching themes as well as various sub-themes emerged from the data analysis. Theme 1 – Care-related challenges linked to the mineworkers, had six sub-themes, namely non-adherence to treatment because of multiple factors, denial and poor understanding of the chronic condition, the lack of knowledge regarding treatment, influence of culture religious beliefs, perceived side effects of medication and mineworkers’ personal choice. Theme 2 – Challenges related to the provision of service to mineworkers and had four sub-themes, namely limiting appointments and consultation times, the lack of tracking and monitoring systems, restrictive and unsupportive working environment, and electronic system limitations (see Table 2).

**TABLE 2:** Themes and sub-theme that emerged.

Themes	Sub-themes
1. Care-related challenges linked to the mineworkers	1.1 Non-adherence to treatment because of multiple factors 1.2 Denial and poor understanding of the chronic condition 1.3 The lack of knowledge regarding treatment 1.4 The influence of culture and religious beliefs 1.5 The perceived side effects of medication 1.6 The mineworkers’ personal choice
2. Challenges related to the provision of service to mineworkers	2.1 Limiting appointments and consultation times 2.2 The lack of tracking and monitoring systems 2.3 Restrictive and unsupportive working environment 2.4 Electronic system limitations

#### Theme 1: Care-related challenges linked to the mineworkers

Occupational health practitioners suggested that they do have challenges providing care to the mineworkers diagnosed with chronic illnesses. Most of the participants’ explanations agreed in principle that they offer health services. However, there are still mineworkers who can stay for months defaulting from taking treatment. In addition, they indicated that mineworkers diagnosed with chronic illnesses play a crucial role in non-adherence to the collection of medication. Mineworkers diagnosed with chronic illnesses would choose not to visit the clinic for follow-ups or to collect their medication as prescribed on their cards. Moreover, mineworkers would choose ‘joining’ (*meaning to work overtime*) as they want to get money and neglect their health needs. Participants identified mineworkers as the defaulter to collecting their medications:

‘I don’t know how they managed to dodge the system because we can easily block.’ (Participant 8, 46 years old, male, ON)

‘So, the employees are not coming by themselves. Sometimes it’s not managers who say they must do the ‘joining’ [*meaning to work overtime*] because you talk about the overtime.’ (Participant 6, 45 years old, female, ON)

‘We always write date of collection on their cards and give them time when they must come and collect their medication but that is not helping as some don’t come for collection.’ (Participant 2, 36 years old, male, ON)

**Sub-theme 1.1: Non-adherence to treatment because of multiple factors:** Although they provided healthcare services to mineworkers diagnosed with chronic illnesses, non-adherence was observed among some mineworkers. In some instances, mineworkers would be diagnosed with chronic illnesses and given treatment; however, after one or 2 months or even years an employee would choose not to return for follow-up or check-up and collect their prescribed medication. This would result in non-compliance and some mineworkers having uncontrolled conditions, which could result in primary and secondary complications. Most participants reported non-compliance of medication as the challenge:

‘[*W*]e encounter it as the patients don’t take their medication.’ (Participant 1, 47 years old, female, ON)

‘[*E*]mployees’ default from taking medication as well as visiting the clinic.’ (Participant 4, 46 years old, female, ON)

‘Yoh the main challenge is compliance.’ (Participant 2, 36 years old, male, ON)

‘So, sometimes you’ll have somebody that has defaulted decided to stay away from treatment for many, many years.’ (Participant 3, 52 years old, female, ON)

‘[*T*]he challenge for me will be the employee not compliant on taking his treatment.’ (Participant 5, 50 years old, female, ON)

**Sub-theme 1.2: Denial and poor understanding of the chronic condition:** Occupational health practitioners suggested that some mineworkers present with denial and poor understanding of their chronic illnesses. Mineworkers diagnosed with chronic illnesses would indicate that they do not believe that they have the condition even after being diagnosed. In addition, the stubborn behaviour has been reported even though it was by few participants. It is worth stating that in this study:

‘[*T*]hey’re still in some state of denial in terms of the treatment.’ (Participant 3, 52 years old, female, ON)

‘It’s a pity mine employees are stubborn.’ (Participant 1, 47 years old, female, ON)

‘[*S*]ome of them they refuse to accept that they have this illness.’ (Participant 4, 46 years old, female, ON)

**Sub-theme 1.3: The lack of knowledge regarding treatment:** The findings suggested that some mineworkers lack knowledge regarding the benefits of their treatment including the side effects. Level of education was reported as one factor which plays a crucial part in mineworkers’ comprehensive understanding of the information and health education provided to them. Participants reported that their pre-knowledge from their communities about certain conditions affect the information and health education given to them at the clinic:

‘[*S*]ome will tell you they didn’t know that the medication should be taken continuously.’ (Participant 1, 47 years old, female, ON)

‘[*A*]t the same time, mine employees have low levels of education. They will be having that information that they got from their communities and sometimes from their colleagues, from their mine.’ (Participant 6, 45 years old, female, ON)

‘[*L*]iteracy affect them as some things do not understand them well.’ (Participant 2, 36 years old, male, ON)

**Sub-theme 1.4: The influence of culture and religious beliefs:** Occupational health practitioners revealed that some influences of culture and religious beliefs impact adherence to medication by mineworkers. The participants indicated that mineworkers preferred to use muti or herbs for their chronic conditions rather than the medication provided at the clinic as they feel and believe it affects and lowers their sexual drive. In addition, it was reported that mineworkers believe some chronic illnesses are demons, which possesses them, and it can be cast or outcasted by their church pastor:

‘[*B*]ut they will tell you about their culture or religion which is better than these medications. and they mostly say this medication affects their erections, or they will say it affects their libido or sex drive in general.’ (Participant 2, 36 years old, male, ON)

‘[*S*]ome do say that, but it is mostly traditional and religious beliefs, there is an old man who came with hypertension and said not this is just a spirit one day I will cast it out it will be gone.’ (Participant 9, 34 years old, male, Social Worker)

‘Remember even with culture we different and they can buy in on anything especially with the talk of manhood thing some say eish I can’t take this pill as it kills me at home [*sexually*].’ (Participant 7, 42 years old, female, ON)

**Sub-theme 1.5: The perceived side effects of medication:** Furthermore, it was echoed by occupational nurses that mineworkers have perceived ideas about side effects brought by medication prescribed for their chronic illnesses:

‘[*T*]o think that they’re not going to perform sexually when they’re taking the treatment not knowing.’ (Participant 3, 52 years old, female, ON)

**Sub-theme 1.6: Mineworkers’ personal choice:** The participants also emphasised that employees choose not to comply with taking medication, visiting the clinic and check-ups at the clinic, even when they have the return date. It is suggested by most participants that mineworkers prefer working overtime and make more money compared to visiting the clinic for the collection of medication as well as taking care of their health. In addition, participants cited that mineworkers mentioned that their supervisors want production as the main focus. Furthermore, it was reported that even when they knock off early or they are off duty they choose not to visit the clinic for check-ups and collection of medication:

‘[*T*]hey choose to take overtime and forget to come to the surface to the clinic.’ (Participant 3, 52 years old, female, ON)

‘[*I*]t is hard at time to convince employees to come to the clinic as they always talk about their supervisor and production. They normally say when they come to the clinic it affects production and they are unable to do joining [*overtime*] as they affect their team by production.’ (Participant 1, 47 years old, female, ON)

‘[*W*]e found out that most of them complain about their overtime [*joining*], which requires them to do extra hours, some make such requests but still do not go and collect their medication because they are more focused on production.’ (Participant 2, 36 years old, male, ON)

‘Problem is. They choose to work overtime. So even when they knock off earlier, they choose to do an overtime.’ (Participant 6, 45 years old, female, ON)

#### Theme 2: Challenges related to the provision of service to mineworkers

The provision of service is hampered by several factors as cited by participants, such as the long queues and slowness at the mine clinic which discourage and prevent the mineworkers from visiting the clinic. The ratio of mineworkers serviced at the clinic compared to the (occupational nurses) ONs is too high and results in compromised service. It is suggested that some mineworkers opt to seek help at their nearest clinic where they reside because of poor service on account of the long queues. Furthermore, it was stated that mineworkers lack support from their supervisors who focus on productivity rather than the good health and well-being of the mineworkers, resulting in them defaulting in collecting their medications:

‘[*A*]nd some of them they stay far from where they work and the lines become long.’ (Participant 3, 52 years old, female, ON)

‘[*W*]e have a large number of employees so it’s easy for them to be missed more especially the diabetes and hypertensive.’ (Participant 8, 46 years old, male, ON)

‘Even from their work section they are not getting support from their immediate supervisor.’ (Participant 2, 36 years old, male, ON)

**Sub-theme 2.1: Limiting appointments and consultation times:** The participants revealed that the operating hours at the clinic are not aligned with the working hours and shifts of the mineworkers whom they service. It is indicated that mineworkers would knock off or end their shift at 04:00 am and are expected to wait for the clinic to open at 07:00 am to collect their medication or come for a check-up. This non-alignment of clinic hours with working hours discourages collection of medication in most mineworkers as they knock off exhausted and cannot be expected to wait for hours for the clinic to open. Occupational nurses suggested that the appointment for annual medical surveillance are limited. Mineworkers are consulted either after 3 or 6 months depending on the referral of Occupational Medical Practitioner. Chronic illnesses can develop or become more complicated during the space of 12 months. Furthermore, this encourages non-compliance from mineworkers which impacts on their health:

‘So, the poor patients, they wait for us from 04:00 until 7:00 to collect that. So, it is strenuous for them.’ (Participant 3, 52 years old, female, ON)

‘[*W*]e only see them once in a year.’ (Participant 1, 47 years old, female, ON)

‘At times they will say when they come for collection, I would be gone, or the pharmacy will be closed.’ (Participant 6, 45 years old, female, ON)

‘[*T*]hen I make a script for 3 months and for the next 2 months patients will be collecting their medication not coming to me.’ (Participant 2, 36 years old, male, ON)

**Sub-theme 2.2: The lack of tracking and monitoring systems:** Occupational health practitioners suggested that tracking and monitoring systems are in place but keep limited information and that results in most mineworkers being missed after being diagnosed. The participants reported that all information is written in the employee’s file and their card which includes return dates. However, mineworkers can be missed until they go for medical surveillance which is conducted once a year as there is no system that reflects their next visits. In addition, it was reported that the current health monitoring system has limited access. The ONs working in primary health section, chronic section, can view only certain information about the health of the employees. The limitation on access to the system hampers service delivery and comprehensive treatment of the employee:

‘What I mean is that the system is basically designed in such a way that we are not able to pick up who is defaulting.’ (Participant 2, 36 years old, male, ON)

‘So, it does not have all our patients on the system. So, sometimes you will have someone that has defaulted and decided to stay away from treatment for many, many years.’ (Participant 3, 52 years old, female, ON)

‘We do not have system showing us that they have skipped collecting their medication.’ (Participant 6, 45 years old, female, ON)

‘The ones that the system would not pick up.’ (Participant 8, 46 years old, male, ON)

**Sub-theme 2.3: Restrictive and unsupportive working environment:** The participants revealed that the working environment is restrictive and unsupportive to mineworkers diagnosed with chronic conditions and that can aggravate the work-related problems and facilitate unsustainable employment. In addition, it was cited that mineworkers are threatened with being replaced by fit employees when they mention visiting the clinic for the collection of medications or check-ups. They indicated that addressing the identified barriers is crucial to provide support to affected mineworkers who compromise their health. One participant indicated that the support that mineworkers have is access to the hotline:

‘I don’t think there is any solid support we have compared to the hotline.’ (Participant 9, 34 years old, male, Social Worker)

‘When employees are supposed to come and collect their medication, their team members tell them that they don’t want to work, and they will have to find someone who is willing to work.’ (Participant 2, 36 years old, male, ON)

‘Is the job itself their job? The job is that because they say to me, sister, when you haven’t finished your job, you can’t leave your station.’ (Participant 3, 52 years old, female, ON)

**Sub-theme 2.4: Electronic system limitations:** The participants echoed that there are limitations related to electronic systems implemented at the clinic for monitoring the mineworkers’ conditions and services offered. They indicated that access to the system is limited as it is not integrated into all departments and the practitioners in the clinic cannot view all the employees’ comprehensive health information. In addition, they all agreed that these challenges compromise the quality of care offered and the overall well-being of the mineworkers as they need to be treated holistically. The participants suggested that functional integrated electronic systems with efficient alert mechanisms that remind ON’s about upcoming employee appointments, collection of medications and those diagnosed with chronic illnesses will drastically reduce non-compliance:

‘Yes, there are certain things that primary health care team cannot see on the system that can only be seen by the chronic illness team on the system.’ (Participant 1, 47 years old, female, ON)

‘So here the system that we are using it cannot pull out a report to say for this month we are expecting so and so to come for their appointment and so and so missed their appointment.’ (Participant 2, 36 years old, male, ON)

OR ‘The system is not able to do that which could help us in advance because we should be able to see in a system.’ (Participant 2, 36 years old, male, ON)

‘Then they come back today when we capture him on Q-Med, capture that person as if it is new because they never know that they knew that patient before. we do not, our system does not pop up to say today Mma Gauta [*meaning Mrs Gauta*] is supposed to come, she is not here like this and that.’ (Participant 3, 52 years old, female, ON)

## Discussion

The aim of the study was to describe factors contributing to the non-adherence of medication among mineworkers diagnosed with chronic illness at a selected clinic according to the ONs. High quality health system is a valuable source that contributes to healthy individuals and financial security (Kruk et al. 2018:1196; Lingard & Turner 2015:18). Employees are expected to be healthy and fit to ensure they have employment, which would ensure they care for their families. However, some employees find themselves being diagnosed with chronic illnesses, which impact their performance at their workplace.

Chronic illnesses are defined as any mental or physical conditions that can persist for more than a year requiring continuous treatment. The USNCHS (n.d.) states that chronic diseases last for more than 3 months and compromise the social and physical functionality, the quality of life (Centers for Disease Control and Prevention [CDC] n.d.) which impacts on the economic stability of healthcare. Chronic illnesses are defined as any illness which have one or more of these features: need distinct training of the patient for rehabilitation, are caused by non-reversible pathological alteration, they are permanent, leave residual disability, or may be expected to require a long period of supervision, monitoring or attention (WHO 2003).

The participants indicated that mineworkers have at least two comorbid illnesses and are on an average of three prescription of medicines to manage their conditions. Some mineworkers default to collect their medication until they are identified during their annual medical surveillance. The descriptions of the ON’s suggest that there is high non-adherence to taking medication among mineworkers diagnosed with chronic illnesses. Adherence is a main contributing factor to the efficacy of the treatment, while poor or non-adherence reduces ideal clinical value. In addition, adherence enhances the efficacy of interventions intended to advance healthy lifestyles among sick individuals. The WHO (2003) defines non-adherence as the degree to which a person’s actions towards following a diet and/or implementing lifestyle transformations, taking treatment, relates with agreed references from their nurse, therapist, dietician or doctor. Shahin, Kennedy and Stupans (2019:2164) indicated that globally, individuals diagnosed with hypertension and not adhering to their medication are between 20% and 50% of the population. Lemay et al. (2018:1687) state that contributing factors with undesirable beliefs towards medications involved marital status, nationality, lower levels of education and increased illness seriousness, while age and increased illness seriousness were linked to lower medication adherence. It is evident that unmarried employees, refugees, lower academic achievers (Lemay et al. 2018:1689) and the severity of illness and age have a higher impact on medication adherence as indicated by the participants. Kleinsinger (2018:1) holds the same opinion that potential barriers to adherence to medication are intellectual impairment, substance abuse or alcohol use, denial, cultural issues, alternative belief systems and low level of education. On the other hand, Fernandez-Lazaro et al. (2019:3) alluded and suggested that potential barriers to adherence to medication are the highest level of education, level of treatment of the information received, lifestyle behaviour such as alcohol consumption, immigration status, household income, living status, gender and age.

The study results concur with the findings by Aljofan et al. (2023:9) that factors that significantly affects medication adherence can include patient’s educational background, their comorbidities, gender and age. Mineworkers diagnosed with chronic illnesses need to adhere to their prescribed medication to keep their conditions controlled. The participants stated various reasons why mineworkers default from taking their prescribed medications and this reflected their interest in trying to mitigate the issue of non-compliance. The mining sector requires employees who are healthy and fit to perform their jobs optimally, hence the provision of occupational and primary health care services to mineworkers. This study showed that the targeted mineworkers diagnosed with chronic illnesses defaulted and non-adhered to taking their medication which contributed to high rates of non-compliance.

### Impact of chronic illnesses

The WHO (2020) indicated that globally, chronic diseases remain the major cause of disability and death. In 2002, South Africa had 28% of all deaths related to chronic diseases. The chronic illnesses have increased over the years and globally, about 28% of employees are diagnosed with chronic illnesses (Vooijs et al. 2018:26) and this hampers their productivity. The WHO (2020) indicated that about 60% of all deaths globally emanate from chronic illnesses. The most striking observation that emerged in Denmark in 2013 was that about 35% of employees were diagnosed with at least one chronic disorder (Mehta et al. 2022:560). Zhang et al. (2024:1543) draw our attention to an early onset of T2D in Chinese patients when it was discovered that they experience stress from life or work. It is evident that some chronic illnesses that employees are diagnosed with emanate from their work environment or from their personal lives including their family stresses. Meng, Robinson and Smith (2017:296) suggested that employees who are diagnosed with chronic illnesses can experience several barriers towards taking care of their health including complying with medication, which results in stress. These barriers can be because of the lack of adequate health information (Aljofan et al., 2023:7), lack of support from family members, expensive medication and lack of support from their colleagues and their employers.

### Complications of chronic illnesses

#### Diabetes and hypertension

Employees diagnosed with chronic illnesses who do not adhere to taking their medication results in high changes of having acute and chronic secondary complications (Yu et al. 2023:3). Chronic secondary complications vary depending on the chronic illness. Employees diagnosed with T1D and T2D who do not adhere to their prescribed medication can result in having acute complications such as hyperglycaemia, diabetic ketoacidosis (DKA), hypoglycaemia and diabetic coma (Farmaki et al. 2020:249). Chronic complications of T1D and T2D are but not limited to liver damage, diabetic retinopathy, diabetic foot, macroangiopathy, osteoporosis, vulnerability to infections, diabetic nephropathy, myopathy and arthropathies (Farmaki et al. 2020:250). It is vital that employees diagnosed with diabetes mellitus type 2 take their medications as prescribed because unmanaged diabetes mellitus has long-term negative effects on all organs in the body (Farmaki et al. 2020:250). Furthermore, Kansra, Calvert and Jones (2021:4) indicated that individuals diagnosed with T2D before the age of 45 suffer a heightened albuminuria risk, particularly males. The other chronic illness, hypertension, which Yu et al. (2023:2) indicated that at least one individual globally receiving hypertensive medication out of three has a controlled blood pressure. Hypertension can have several complications such as heart valve disease, coronary artery disease, kidney disease, atrial fibrillation and either diastolic or systolic heart failure (Fuchs & Whelton 2020:287) and (Rosenfeld et al. 2022:1).

#### Non-adherence to treatment because of multiple factors

Some mineworkers were noticed by ONs to be avoiding and defaulting from the prescribed medication. Various motives for non-adherence were mentioned by participants and what can be the contributing factors among mineworkers diagnosed with chronic illnesses. The WHO (2003) indicated that approximately 50% of individuals diagnosed with chronic illnesses in developed countries adhere to long-term treatment, while in developing countries the adherence rates are lower, which reflects that individuals struggle with compliance and adhering to prescribed medication and therapies. Fernandez-Lazaro et al. (2019:6) indicated that the other factor to non-adherence among 70% of individuals diagnosed with chronic illnesses were that they tend to forget to take treatment occasionally. All employees including those diagnosed with chronic illnesses are allowed and expected to visit clinics when they are sick and also for follow-ups including for collection of their medications. However, some employees diagnosed with chronic illnesses choose not to visit the clinic for follow-up including not collecting their medications as prescribed by the occupational health nurse or the doctor. The mineworkers who choose not to adhere to taking their medications as prescribed results in their conditions deteriorating and having secondary complications. Cheen et al. (2019:2) alluded that challenges that keeps recurring that undermine the effectiveness of the healthcare system is non-adherence to medication by individuals who are diagnosed with chronic illnesses. When individuals fail to collect their medication, as well as not complying to follow-up visits, it results in them becoming sick. (Yu et al. 2023:4), overcrowding of hospitals and the chronic illness not being controlled. Kansra et al. (2021:4) stated that adherence to effective treatment and medication such as inhalers, injections and tablets is challenging and the significant role and view about adherence is played by the patient. In previous studies, Jin, Kim and Rhie (2016:2119); Mehari, Giorgis and Shibeshi (2016:4); and Al-daken and Eshah (2017:266) suggested that one of the main factors that contributed to the high rates of non-adherence to taking medication is the medication’s frequency. In recent studies, non-adherence to taking medication by employees with chronic illness can be caused by various factors, such as the high costs of medication, the lack of belief in the use of the medication, the lack of knowledge about the illness, side effects brought by treatment as well as cultural and religious beliefs (Aljofan et al. 2023:1).

#### Denial and poor understanding of the chronic condition

Denial is defined as a specific intellectual mechanism, a psychotic-like twist of reality, a coping mechanism and evidence of neurological impairment to which can be an add-on phase of intellectual adaptation following being diagnosed with a chronic illness and a multiple set of psychological and satisfying processes that attempt to disable external and internal stimuli (Livneh 2009:227).

When employees do not have extensive information about their chronic illnesses, their health effects and the health services available, they will continue with habits that aggravate their health and are unable to access care timeously. The participants associated denial and poor understanding of the chronic condition with non-adherence to treatment as the contributing factors among the employees diagnosed with chronic illnesses. The participants indicated that employees present with denial after being diagnosed with chronic illnesses. There are various types and patterns of denial. When information about employees’ condition is disclosed to them, they start to function at different levels of awareness.

It is believed that some employees have denial after being diagnosed with chronic illnesses, while others have a poor understanding of their conditions because of their level of education. Chimezie (2023:69) defines health literacy as general awareness and knowledge about health, health basics, healthcare services, illnesses, promotive and preventative measures. Park et al. (2018:176) indicated that for healthcare professionals to increase adherence to medication, it is critical that they understand the fundamental cause of non-adherence among individuals. In addition, it is crucial that individuals diagnosed with chronic illnesses have the ability to discover and comprehend, apply knowledge and services to make informed health-linked decisions and act appropriately for others and themselves. Van der Heide et al. (2018:135) indicated that health literacy signifies the collaboration between patients and/or communities and healthcare providers, employers and/or organisations and healthcare professionals. Chimezie (2023:64) reiterated that health awareness and health literacy are crucial approaches in supporting and enhancing global health and improving access to healthcare services.

#### The lack of knowledge regarding treatment

Occupational health practitioners indicated that the lack of knowledge regarding treatment among employees also impacts non-adherence to medication. Kvarnström et al. (2021:6) alluded that, at times, individuals may have inadequate data or intelligence to comprehend their treatment programme. The participants stated that continuous health education would assist employees to adhere as they would be having a better understanding. It is alluded that to encourage better adherence, it is critical for employees to comprehensively understand their chronic illnesses as well as a robust belief about the medication prescribed (Park et al. 2018:180). Fernandez-Lazaro et al. (2019:6) indicated that the other factor for non-adherence among 21.1% of individuals diagnosed with chronic illnesses was that they tend to stop taking their treatment when they feel better. In Hong Kong, they provide about 70% of chronic disease care in their health services to increase compliance among individuals diagnosed with chronic illnesses (Yu et al. 2023:2). As medication plays a vital role in the management and treatment of various chronic illnesses, it is critical to understand factors causing non-adherence of medication. Health practitioners often underestimate the ‘burden of treatment’ experienced by individuals diagnosed with chronic illnesses (Kansra et al. 2021:2). Occupational health practitioners are to ensure mineworkers’ knowledge of their condition and treatment. Health care providers are to provide continuous health education to individuals diagnosed with chronic illnesses. The formation of support groups can assist as individuals diagnosed with chronic illnesses have different experiences and they can share with others.

#### Influence of culture and religious beliefs

The results of this study also suggest that employees support culture, religion and witchcraft as prevalent causal beliefs. The participants indicate that employees believe that some illnesses are caused by evil spirits and can be outcasted by their pastor at church. Becker et al. (2019:4) alluded that participants believe that mental illness occurs ‘when the trees blossom’ and individuals will go back to normal after the change of season. The participants’ perspectives highlighted that the employees’ beliefs about medication influence their behaviour towards medication adherence thus influencing whether they contribute to the committed role in managing their conditions. Horne et al. (2013:2) and Lemay et al. (2018:1687) alluded that the increased undesirable beliefs of patients about medication persuade medication commitment and adherence.

#### Perceived side effects of medication

All pharmaceutical medication has different types of undesired or unexpected and unpredicted medicine-induced effects. Other side effects of medication can have a significant detrimental effect on the individual’s daily living. According to Edwards and Aronson (2000:1255), the effects of medication can be referred to as side effects as they can be accompanied by beneficial and damaging therapeutic consequences. Individuals who have a prescription of medication are to adhere to prescribed treatment to ensure compliance and to control conditions. There are certain coping behaviours that would be displayed by individuals who are expected to comply with their treatment. Coping mechanisms can have an impact on how individuals react to adverse drug responses (ADRs), as well as their recovery to their conditions. The ADRs are one insistence in which coping style may influence behaviour of individuals diagnosed with chronic illnesses (O’Donovan et al. 2019:1). Comprehension regarding the importance of complying with the treatment even when they experience ADRs is crucial.

In addition, consulting healthcare professionals for another alternative of medication is vital when ADRs are considered as undesirable. Närhi and Helakorpi (2007:51) suggested that appropriate facts about drugs can result in improved adherence and treatment results. Fernandez-Lazaro et al. (2019:6) indicated that another factor to non-adherence among 24.1% of individuals diagnosed with chronic illnesses was that they tend stop taking their treatment when they feel worse after taking medication. The participants indicated that the employees chose to stop medication without consulting the ON’s or the occupational medical practitioners, even when they were given health education to report any undesirable feelings while taking medication. It is evident that employees need to be educated about their conditions and reasons for prescribed medication, including the side effects of the medication. Previous research indicated that 79% of participants had physical side effects while 11% had psychological impacts brought by their prescribed medication (O’Donovan et al. 2019:2). Even though individuals have been informed of the side effects of their medication, they tend to forget and opt to stop taking their medication when they have ADRs. In addition, healthcare professionals are not easily accessible when individuals experience ADRs. Setting-up a hotline can help individuals when they experience undesirable and unexpected side effects.

#### Employees’ personal choice

Fernandez-Lazaro et al. (2019:6) indicated that the other factors to non-adherence among 29.3% of individuals diagnosed with chronic illnesses was that they tend to be careless about taking their treatment. The lack of adherence to medication contributes to the high burden on the healthcare system as employees become sick, absent themselves from work and stand in long queues at the clinic. The results suggested that organisations have to play a role in improving the health of employees; however, employees need to be responsible about their conditions. Brown and Bussell (2011:304) suggested that after consultation with patients, they can recall approximately 50% of what the pharmacist discussed. Employees are to display interest in their conditions and be proactive in requesting for assistance and care. Self-care is promoted as it is considered a main point for patients with chronic illnesses. Healthcare providers are to support patients in self-care as it is a crucial factor in caring for chronic illnesses (Ausili et al. 2014:180).

#### The lack of tracking and monitoring systems

Occupational health practitioners indicated that they find it very difficult to manage this challenge as the mine clinic has non-functional, disintegrated electronic systems, which cannot efficiently alert and remind them about employees’ next check-up or medication collection. This results in most employees being missed and only discovered when their health deteriorates as well as having complications from the condition or during the medical surveillance, which is conducted once a year. Schwarz et al. (2022:2) suggested that intricate pathways and the lack of coordination are the main contributing factors to non-adherence.

#### Restrictive and unsupportive working environment

The participants indicated that the support from colleagues and supervisors is limited as some employees stated that the leader expects them to work rather than to visit the clinic. It is crucial that for the patient diagnosed with chronic illness, the community, including the employer and the family support and play their role actively to ensure the efficient provision of care to the patient (WHO 2020). Bosma et al. (2021:6) indicated that various representatives of the employers have difficulty with supporting employees, and supervisors have no knowledge of laws and regulations that protect employees’ health and safety (Bosma et al. 2021:6). Arnold, Harris and Weale (2023:23) state that employers have the potential to improve the health of employees using working hours, support groups, counselling sessions, supervisors’ practices, levels of supervision and policies. The support would be appreciated by most employees as they spend most of their time at the workplace. However, most employers focus on productivity and ignore the health of employees. Kolmet, Mariño and Plummer (2006:83) suggested that the working class are at higher risk of developing chronic illnesses and poorer health compared to managerial and administrative workers.

### Study’s limitations

The generalisation of this research findings cannot be applied to employees working in the mining sector as the study was conducted in one mine clinic and one province in South Africa. These are the views of ONs and exclude other participants who took part in the main study.

### Study’s recommendations

Further studies are required on non-adherence to taking medication by employees diagnosed with chronic illnesses. Occupational health practitioners indicated that they could take measures to encourage employees to adhere to medication. These measures would include providing continuous health education and health campaigns to employees diagnosed with chronic illnesses.

## Conclusion

The study was justified by its purpose of describing factors contributing to non-adherence to medication among mine employees diagnosed with chronic illnesses according to ONs. The research results indicated that the participants noted non-adherence to medication among mine employees diagnosed with chronic illnesses.
